# Idiopathic aggressive myositis ossificans of the hand infiltrating the flexor sheath/checkrein ligament, obliterating the common digital artery, and attenuating the digital nerve: A case report

**DOI:** 10.1016/j.ijscr.2018.11.042

**Published:** 2018-11-22

**Authors:** Maha Arafah, Mohammad M. Al-Qattan

**Affiliations:** a Department of Pathology, King Saud University, Riyadh, Saudi Arabia; b Department of Surgery at King Saud University, Riyadh, Saudi Arabia; c King Faisal Specialist Hospital and Research Center, Riyadh, Saudi Arabia

**Keywords:** Myositis ossificans, Hand, Infiltrating tumor

## Abstract

**Introduction:**

Myositis ossificans of the hand is extremely rare and tumors do not usually infiltrate adjacent structures. We present a very unusual case with a tumor infiltrating adjacent structures.

**Report of a case:**

A 30-year-old female presented with a 5-week history of a painful rapidly-growing mass over the proximal phalanx of the right ring finger. The mass infiltrated the entire flexor sheath over the proximal phalanx as well as the checkrein ligament of the proximal interphalangeal joint. The common digital artery of the 3rd web space was obliterated by the tumor. The radial digital nerve of the ring finger was stretched and attenuated. Total excision was done and histology confirmed the diagnosis of myositis ossificans. No recurrence was seen at the one-year follow-up.

**Discussion:**

Myositis ossificans of the hand is extremely rare. We report our case to document the unique feature of tumor infiltration of adjacent structures. We also document that excision is curative despite the infiltrative nature of the tumor.

**Conclusion:**

Our case is unique because of the infiltrative nature of tumor. There is no indication for amputation even for infiltrative tumors. We demonstrate that complete excision is curative with good regain of function even in infiltrative lesions.

## Highlights

• Myositis ossificans of the hand is extremely rare.
• These tumors do not usually infiltrate adjacent structures.
• We present a case with infiltration of adjacent structures.
• Complete excision was curative.

## 1 Introduction

Myositis ossificans is a benign form of heterotopic ossification. It usually arises from skeletal muscles and present as a painful soft tissue mass with overlying skin erythema. It is most frequently seen in the thighs of active athletes. The two commonly involved muscle groups are the quadriceps and adductor muscles of the thigh. Most cases resolve with conservative management (rest and non-steroidal anti-inflammatory medications) [1].

Myositis ossificans of the hand is extremely rare, and hand tumors have a different presentation and prognosis from the classic thigh lesions. Pain is usually more severe in the hand and this may be related to space limitations within different hand compartments. Furthermore, the proximity of nerves may lead to nerve compression. Finally, most hand tumors require surgical excision despite the initial trial of conservative management either because of intractable pain or persistence of the mass [2]. Simple excision is usually curative because the tumor mass does not usually infiltrate adjacent structures [2].

We report for a very unusual case of myositis ossificans of the hand with infiltration of the flexor sheath, checkrein ligament of the proximal interphalangeal join, and the neurovascular bundle. The work has been reported in line with the SCARE criteria [3].

## 2 Case report

**Fig. 1:**
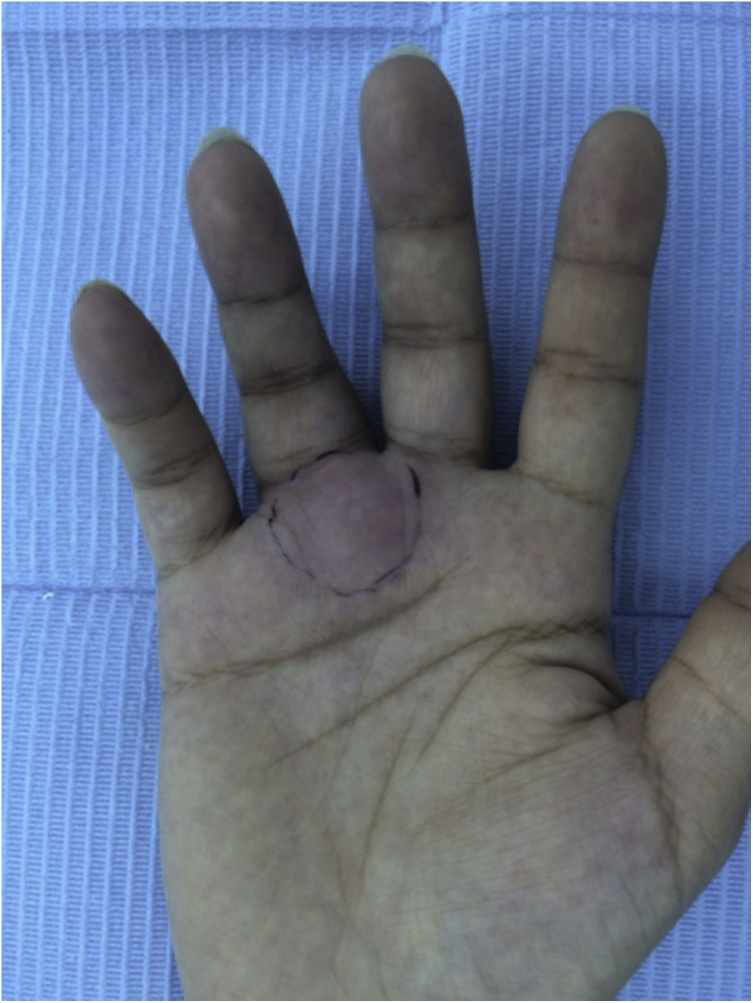
Preoperative appearance showing the mass and the overlying skin erythema.

**Fig. 2:**
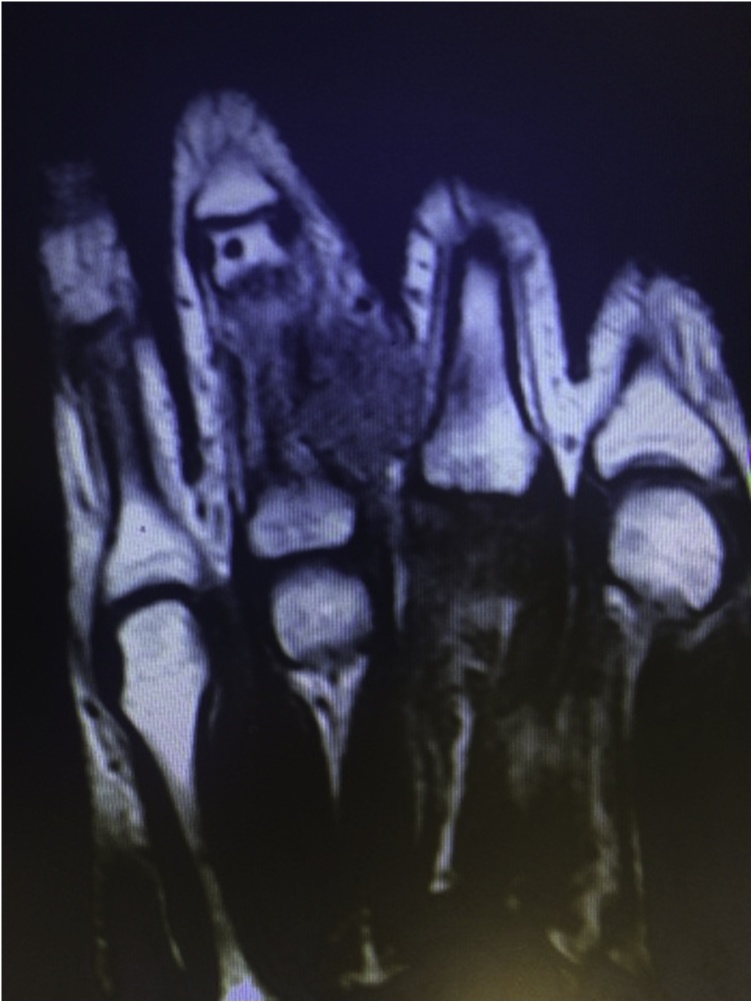
Magnetic resonance T1- weighted image showing an *iso*-intense lesion.

**Fig. 3:**
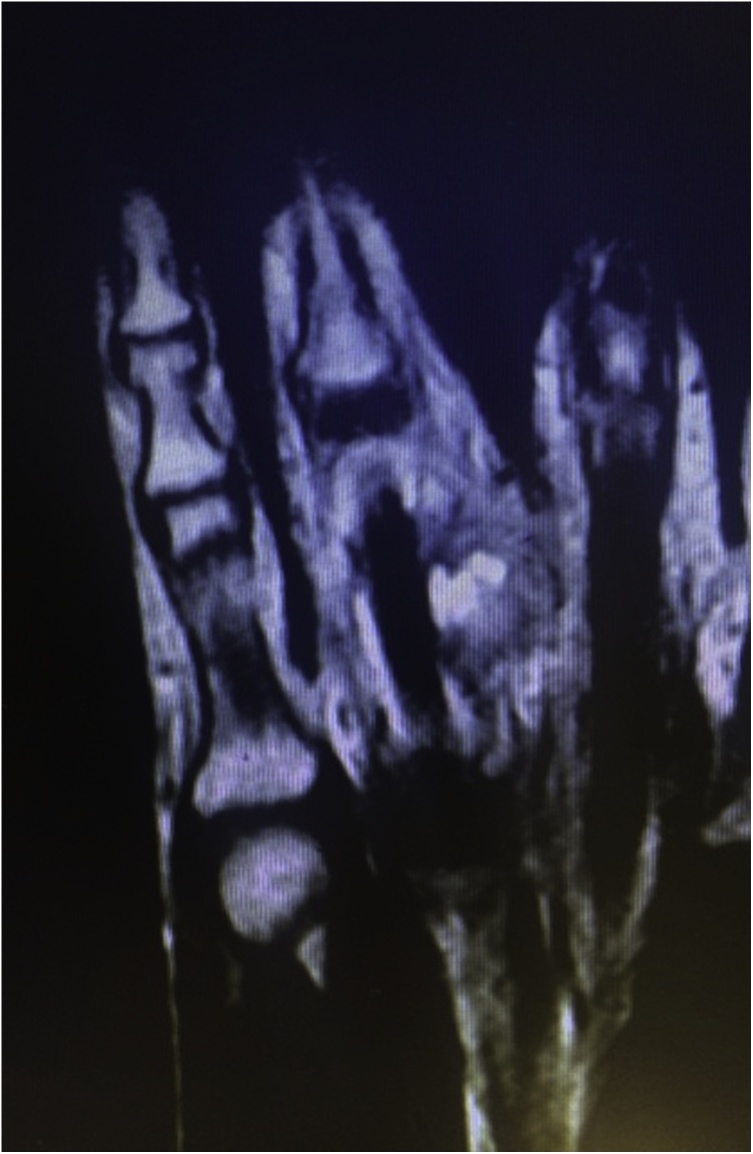
Magnetic resonance T2- weighted image showing high intensity in the center and low intensity at the periphery of the lesion.

A 30-year-old female presented with a 5-week history of a painful rapidly-growing mass over the proximal phalanx of the right ring finger (Fig. 1). The patient also complained of numbness along the distribution of the radial digital nerve of the finger. There was no history of trauma. Examination showed a firm tender mass measuring 3.5 × 2 cm with an overlying skin erythema. The patient was unable to fully extend or flex the finger because of pain. There was also a flexion contracture of the proximal interphalangeal joint. Static two-point discrimination on the radial side of the ring finger was 10 mm. Plain x-rays showed no bony abnormalities or calcifications. Magnetic resonance imaging showed an *iso*-intense (similar to intensity of muscle) lesion on T1-weighted images (Fig. 2). T2-weighted images showed high intensity in the center of the lesion and low intensity at the periphery of the lesion (Fig. 3).

**Fig. 4:**
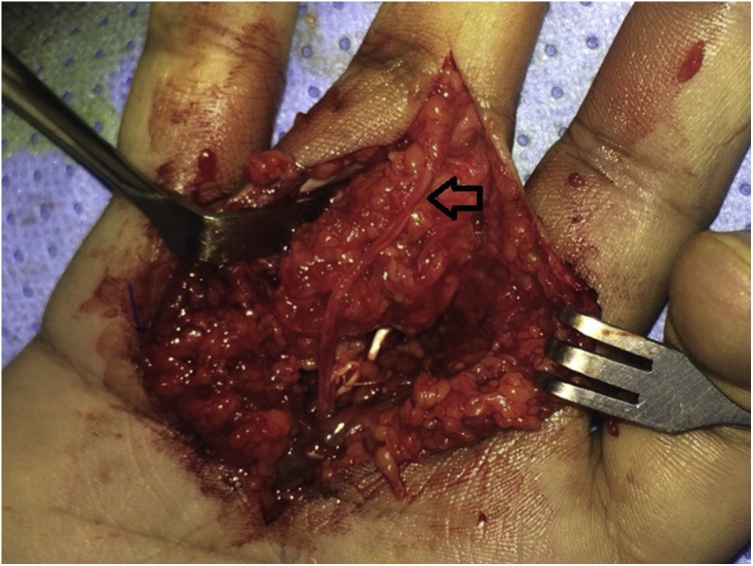
Intraoperative view of the lesion. The flexor sheath and the checkrein ligament of the proximal interphalangeal joint were infiltrated by the tumor. The common digital artery of the 3^rd^ web space was obliterated (within the tumor). The digital nerve was stretched and attenuated (arrow).

**Fig. 5:**
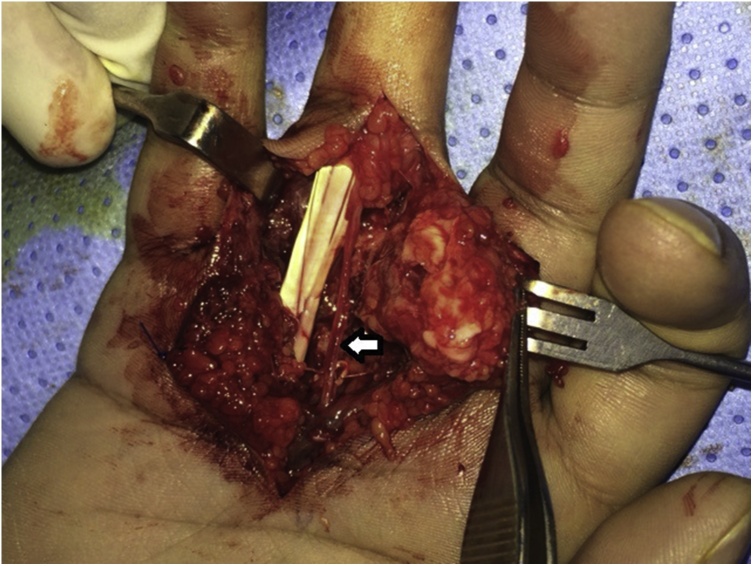
Excision of the mass required excision of the flexor sheath, the checkrein ligament, and the common digital artery. Note the preserved digital nerve (arrow).

**Fig. 6:**
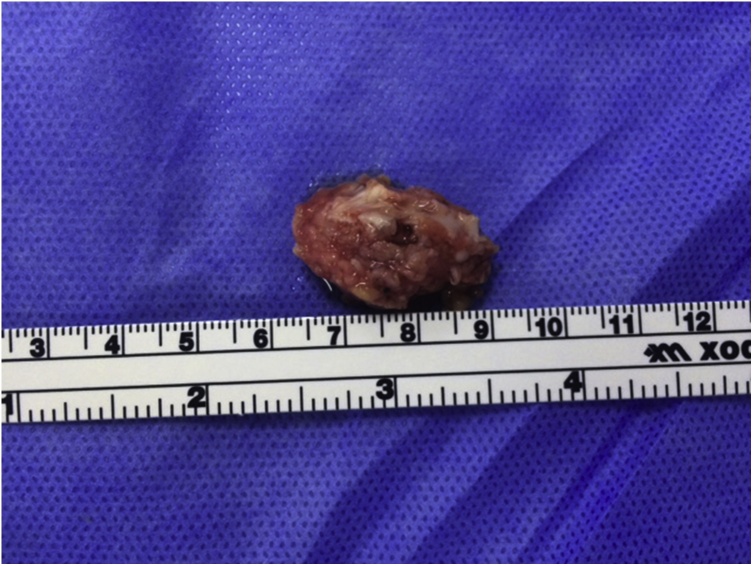
The excised tumor mass.

Surgical excision was done under general anesthesia. The mass infiltrated the entire flexor sheath over the proximal phalanx as well as the checkrein ligament of the proximal interphalangeal joint. The common digital artery of the 3rd web space was obliterated by the tumor. The radial digital nerve of the ring finger was stretched and attenuated (Fig. 4). Total excision was done and this required excision of the flexor sheath over the proximal phalanx (including the entire A2 pulley), the checkrein ligament and the common digital artery. The digital nerve was preserved (Fig. 5, Fig. 6).

**Fig. 7:**
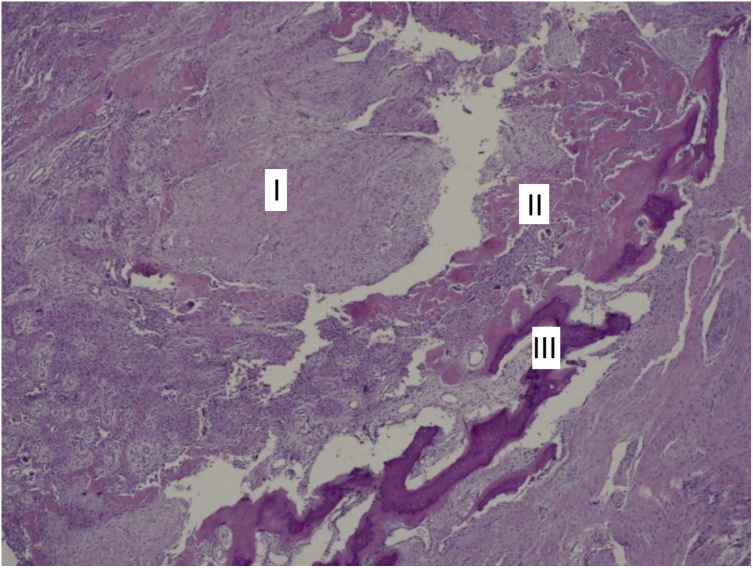
Histology showing the classic 3 zones of myositis ossificans: I: Central cellular fibroblastic zone, II: Osteoid Zone, III: Zone of mature bone trabeculae.

**Fig. 8:**
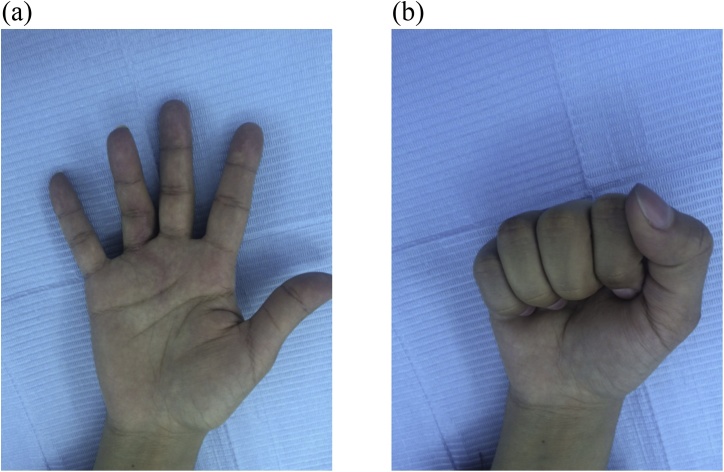
Active motion at 1 year after surgery. A: Active extension, B: Active flexion.

Histology showed the classic 3 histological zones of myositis ossificans: a central cellular / fibroblastic zone, an intermediate zone of osteoid (immature bone) and an outer zone of mature bone (Fig. 7). The patient was followed up for one year after surgery with no evidence of recurrence. At final follow up, there was full flexion of the digit, but the proximal interphalangeal joint had an extension lag of 30^o^ (Fig. 8). Upon palpation, bowstringing of the flexor tendon was felt over the proximal phalanx. The static two-point discrimination on the radial side of the ring finger improved to 6 mm.

## 3 Discussion

Myositis ossificans of the hand is extremely rare. We report our case to document the unique feature of tumor infiltration of adjacent structures. We also document that excision is curative despite the infiltrative nature of the tumor.

We reviewed all previously reported cases of myositis ossificans of the hand and wrist since 1982 [2,[4], [5], [6], [7], [8], [9], [10], [11], [12], [13], [14], [15], [16], [17]]. There was a total of 18 cases (including our case) and their data are summarized in Table 1. Females (n = 11) were more commonly affected than males (n = 7). Five cases were seen in patients 15 years or younger; and the remaining 13 patients were adults (18–58 years old). The majority of patients (n = 16) had tumors within the hand (fingers/web spaces/palm/thenar area) and only two cases had wrist lesions. Three lesions appeared during pregnancy [4,16]. None of the tumors invaded bone, although a periosteal reaction of the adjacent bone was seen in some cases [8,10]. Two lesions arose from the lumbrical muscles [2,17] and three lesions from the thenar muscles [12,14,15]. The remaining lesions (n = 13) did not arise from muscles. Nerve compression was seen in two cases and complete recovery of nerve function was documented in both cases following excision [2,11]. In our case there was a stretching and attenuation of the adjacent digital nerve and sensibility did not return back to normal at 1 year after surgery. Only one previously reported case documented tumor infiltration into the extensor tendon of the little finger [6]. In our case there was infiltration of the flexor sheath, the checkrein ligament and the common digital artery. Only two cases were successfully treated with conservative management [12,13]. Another three cases [[4], [5], [6]] were treated with ray amputation because of suspected sarcoma; and these cases included the case with extensor tendon infiltration [6]. The remaining cases (including ours) were treated with excision with no recurrence.

**Table 1:** A review of reported cases of myositis ossificans of the hand and wrist from 1982 to date.

Authors/Year	Patient age (years) and sex	Site of the tumor	Infiltration of the tumor	Management	Comment
Schecter et al., 1982 [4]	23, F	Middle finger	No	Ray amputation	Lesion appeared during pregnancy. Amputation done because of suspected sarcoma
Schecter et al., 1982 [4]	25, F	Middle finger	No	Excision	Lesion appeared during pregnancy
De Smet and Vercauteren, 1984 [5]	58, F	Middle finger	No	Ray amputation	Amputation done because of suspected sarcoma
Patel and Desai, 1986 [6]	35, F	Little finger	Extensor tendon	Ray amputation	Amputation done because of tendon involvement and suspected sarcoma
Kai et al., 1987 [7]	35, M	Second web space	No	Excision	Failed initial conservative management
De Smet et al., 1994 [8]	27, M	Near the head of the second metacarpal	No	Excision	Bone scan showed increased uptake of the adjacent second metacarpal
De Smet et al., 1994 [8]	42, M	Middle finger	No	Excision	Periosteal reaction of the adjacent middle phalanx
Goto et al., 1998 [9]	18, F	Thumb	No	Excision	
Kusuma et al., 1999 [10]	37, F	First web space	No	Excision	The adjacent index metacarpal had a periosteal reaction
Kaleli et al., 2003 [11]	31, F	Wrist (volar aspect)	No	Excision	The tumor caused compression of the adjacent ulnar nerve
Jayasekera et al., 2005 [12]	15, M	Thenar area	No	Conservative	Lesion resolved slowly over 5 months
Chadha and Agarwal, 2007 [13]	10, M	Wrist (dorsal aspect)	No	Conservative	History of trauma
De Smet and Degraf, 2012 [14]	12, F	Thenar area	No	Excision	History of trauma, initial conservative treatment failed
Akahane et al., 2015 [15]	15, F	Thenar area	No	Excision	Initial conservative treatment failed
Hong et al., 2016 [16]	25, F	Near the 4^th^ metacarpal neck	No	Excision	History of trauma, lesion appeared during pregnancy
Al-Qattan et al., 2017 [2]	38, M	Near the 2^nd^ metacarpal neck	No	Excision	Compression of the radial digital nerve. The tumor arose from the first lumbrical muscle
Monteiro et al., 2018 [17]	5, M	Mid palm	No	Excision	History of trauma. The tumor arose from the third lumbrical muscle
Current case, 2018	30, F	Ring finger^a^	Flexor sheath, checkrein ligament, common digital artery	Excision	Stretching and attenuation of the digital nerve

a The lesion in our case was mostly located along the proximal phalanx of the ring finger, with an extension to the palm and web space.

The MRI features of intra-muscular myositis ossificans is well described in the literature [18]. Early lesions (defined as lesions without calcification; usually in the first 4 weeks) show *iso*-intensity on T1 and homogeneous high intensity on T2 images. A high intensity in the center of the lesion with a low intensity at the periphery of the lesion is a feature of subacute (4–8 weeks) and mature (over 8 weeks) lesions [18]. The MRI of our patient was done at 5 weeks from the appearance of the lesion, which is considered to be in the subacute stage. The MRI findings in our case was consistent with an early lesion on T1 images, and with a more mature lesion on T2 images. This is interesting and may be related that the lesion was not related to muscle.

The most important differential diagnosis of myositis ossificans of the fingers is fibro-osseous pseudo-tumor of the digit (also known as florid reactive periostitis) [19,20]. This tumor is benign but with an aggressive behavior. It is sometimes considered as a superficial variant of myositis ossificans [20]. However, fibro-osseous pseudo-tumors have the following characteristic features: they always arise from the skin and subcutaneous tissue, the histology shows “incomplete” zoning (multinucleate giant cells and myofibroblasts merging with osteoid without clear zones), and the fibroblasts show atypia.

## 4 Conclusions

Myositis ossificans of the hand is very rare. Although an initial trial of conservative treatment is usually tried, most cases end-up with surgical excision either because of intractable pain or persistent mass. The lesion does not usually infiltrate adjacent structures. Our case is unique because of the infiltrative nature of tumor. There is no indication for amputation even for infiltrative tumors. We demonstrate that complete excision is curative with good regain of function even in infiltrative lesions.

## Conflict of interest

None.

## Funding source

None.

## Ethical approval

The study was approved by the research committee, National Hospital (Care), Riyadh, Saudi Arabia.

## Consent

Written informed consent was obtained from the patient for publication of this case report and accompanying images. A copy of the written consent is available for review by Editor-In-Chief of this journal on request.

## Authors’ contribution

Both authors contributed significantly and in agreement with the content of the manuscript. Both authors participated in data collection and in writing of the manuscript.

## Registration of research studies

Not relevant here.

## Guarantor

M.M. Al-Qattan.

## Provenance and peer review

Not commissioned, externally peer reviewed.
